# The Period of Contagiousness of Certain Diseases, and the Best Means of Preventing Transmission

**Published:** 1918-05

**Authors:** E. Swann

**Affiliations:** Merion, Ala.


### BUFFALO MEDICAL JOURNAL

Yearly Volume 73 MAY, 1918 Number 10

### ORIGINAL ARTICLES

**The right is reserved to decline papers not dealing with practical medical and surgical subjects, and such as might offend or fail to interest readers. Contributors are solely responsible for opinions, methods of expression and revision of proof.**

### The Period of Contagiousness of Certain Diseases, and the Best Means of Preventing Transmission.

DR. E. SWANN, Merion, Ala., Capt. M. R. C.

(Published by permission.)

Today the medical profession is spending more money, time, and effort toward prevention of disease, than at any time in the history of the world, and we realize and appreciate the individual who really does something to prevent disease, and the spread of it, far more than the ones who are treating the sufferers who have already contracted it,—the man that can keep the soldier well, and ready to fight, is the one who is worth while.

Pneumonia, while not listed in the textbooks as one of the contagious diseases, is undoubtedly infectious and communicated from one person to another. It is usually spread by immediate contact, either with a person suffering from the disease, or with a convalescent, or healthy carrier. The importance of these modes of transmission varies with the different diseases. With most diseases, however, the healthy carrier may be a greater menace to the community, though the infected individual, because of the failure to detect the carrier condition, as in typhoid and meningitis, is well recognized. It has been proven that a considerable percentage of persons intimately associated with pneumonia patients harbor pneumococci of the same type as those suffering with the disease. It is claimed these carriers harbor the organisms in their mouths for a variable period, averaging 3 to 4 weeks. Another means of conveyance that has been known and recognized for some time is through dust—though very little importance is attached to it, except in rooms where one has the disease. We have considerable evidence, contrary to the opinion previously held, that a good per cent of pneumonia arise by infection from without. Therefore we should have some practical means of prevention. Even if we did not know what the exact cause was, the adoption of such precautions as are taken in the efforts to control the spread of some of our other infectious and contagious diseases would be justifiable. And as we are certain of the essential cause and in a large percentage of our severe cases of pneumonia, even of the mode of transmission, it is very important that we use every means to prevent its spread. To do this in our army hospitals each case should be regarded as a focus for the spread of the infection, and the care of the patient should be such as that of diseases universally recognized as communicable. The patient should be isolated as nearly as is practical, and his communication with others should be reduced to a minimum,—and as we know the pneumococcus finds its way out from the patient, in the secretions from the mouth and lungs, the sputum should all be collected in special containers and burned. Some sputum cups are not wide enough at the top for a sick person to spit in. If they were flared more at the top, there would not be so much danger of the sputum missing the cups, and getting on the clothing, the bedding, or dropping on the floor. Every thing that comes in contact with the mouth should be thoroughly sterilized before using again. The rooms or buildings occupied should be thoroughly cleaned. I don't believe it a good or safe plan, to place many patients suffering with any communicable disease in the same room or too near each other. For several weeks between 30 and 40 cases of measles were crowded in one hospital ward. Several were either accompanied or followed by pneumonia, bronchitis, and catarrhal fever, (pardon the term, but I have no other name at present for the condition), mastoiditis, otitis media and tonsilitis. For a time there was a high mortality in this ward, we were all discouraged at the way things were going. The uncomplicated measle cases were moved into other wards, leaving the pneumonia and ear cases. Afterward there was one death in this ward and it was due to acute tuberculosis. There was an immediate marked improvement of all patients, the old cases began to get better, and there was no further development of pneumonia.

In a ward devoted to mumps, where we did not have so many bunched together, and where the air allowance was abundant, there was only one complication of any consequence—Orchitis, which is part of the original disease. In contrast, a ward crowded with cases of mumps, developed all kinds of catarrhal throat and bronchial conditions. An additional precaution in preventing the spread of disease is the use of bed sheets hung on wires stretched across the room, between each two beds.

The daily cleaning of wards should be done in some way to prevent dust from being disseminated. As I have already stated the pathogenic pneumococci are carried in the mouth and lungs for 3 or 4 weeks, and if space is available it would be a good plan to keep convalescent pneumonias in a hospital until they are strong, and free from pneumococci. I understand this is a difficult problem for the army, and at present not practical, yet I believe it would reduce the number of cases materially. Each convalescent should use the same precaution in daily cleansing the mouth, throat and nose with the same solutions as are used in isolation wards. In concluding this part of the subject I would say, thoroughly isolate, do not crowd wards, separate beds by curtains coming close to the floor—have all attendants take the same precaution as in frankly contagious diseases—determine type if possible, search for carriers, as in other diseases of like nature, and when found isolate them, and I believe we can reduce incidence and mortality of pneumonia considerably..

Measles occurs in wide spread epidemics, being as a rule most common in the spring, though at present there is a world-wide epidemic which began last Sept., and still continues, though abating quite rapidly. It is contagious from the beginning of the catarrhal symptoms, being most contagious during this period, and while the rash is coming out. As the eruption begins to fade, and the catarrhal symptoms clear up, the risk of infection rapidly diminishes. The duration of the infective period is about 3 weeks, or as long as the patient is desquamating or the cough remains. Some few individuals are immune, but I think that number extremely small. The period of incubation is from 7 to 14 days.

The control of measles among our troops, should be studied more closely and more care taken to prevent its spread, for it can be, and is a preventable disease. It should not occur in epidemic form in our army, yet it is killing more soldiers in the aggregate than any other disease. The complications are so numerous and serious, that it is interfering to a great extent with our military efficiency. The cause for this can be removed. One of the most practical measures in controlling the disease, is first to consider it as one of the respiratory diseases, and establish isolation as early as the first symptoms arise, considering of course, any rise in respiratory symptoms as a forerunner of the disease. This precaution must be started in the companies where it first makes its appearance, and cases should be isolated just the same as for meningitis. Look closely after all who are not well; of course this duty falls on the regimental surgeon. One advantage in this method of isolation is that it does not require but 4 or 5 days to find out whether the soldier has measles or not. When a case does break out, all catarrhal cases should be isolated at once. It would be well for the surgeon of each unit to look carefully over each soldier for any respiratory symptoms, Koplik spots, etc. If we wait until the rash appears the infection has had a chance to spread. When a case has once broken out, in about 12 days search for others. It is well to isolate all contacts for 12 or 14 days, ventilate rooms and tents each day, letting in all sunshine possible, and by all means do not allow the soldier to remain in an overheated room. I think it a real neglect of duty on our part that we allow our hospitals in the different cantonments all over the country to be filled from month to month with a preventable disease—especially one that interferes so materially with the training of the soldier.

Mumps is another one of our highly contagious diseases of which no specific organism has ever been found. It is usually communicated by the breath, can be carried by a third person or by fomites; it is most likely to be communicated during the beginning of the attack, but possibly while the febrile symptoms remain. The period of incubation is from 10 to 20 days. The duration of an attack is about a week. Complications outside of orchitis are rare. The patient, and all parties exposed should be quarantined for 3 weeks. At present we are keeping our patients in hospital 3 weeks.

Cerebro spinal meningitis in epidemic form has not proved very contagious among our soldiers.

Flexner states the meningococcus is unknown outside the human host. The organism is kept alive by the human carrier; it enters the body by the mouth, or nose, after which it reaches the central nervous system by way of the lymphatica or blood stream, the former being the more frequent route. Those with a cough are more dangerous than those without. There are four types, type one being the most virulent. The organism multiplies on the mucous membrane. The meninges may become affected soon after the organism reaches the nose or it may not occur for days, weeks, or months thereafter. The danger from a carrier is greater than that from an established and isolated case. The germ may be carried on the bodies of flies. The average duration of the infectivity of a carrier is about four or five weeks. Flexner states that the best method at present of clearing up carriers is to sniff through the nose twice a day a 1% sol. of chloramin with gargling of the throat with the same sol. or a spray of 2% sol. of chloramin T. in oil. The vaccines are of no use in clearing up the throats of carriers, though of considerable value in treatment.

---

**W. H. Morley, Detroit:—The Preparation and Standardization of Ovarian and Placental Extracts.** Surg. Gyn. & Obst. Vol. XXX, 1917. Morley gives due emphasis in his article to the need for more uniform methods in the preparation of ovarian and placental extracts. Tangible laboratory and clinical data are still moreover lacking in extent. A review of the more important articles on the above subject reveals the circumstance that it is only within the last ten years that an attempt has been made to isolate the active principle of the ovary and placenta, especially the former. Iscovesco (1908) obtained "lipoids" from the red blood corpuscles, hypohysis, kidney, adrenals, ovaries, the testicles and the corpora lutae, and discovered they exerted a certain action on the female genitalia. The "homo-stimulating" lipoids, he found, had an action on the same organ from which they were derived, the "hetero-stimulating" lipoids exercising an action on different organs—this division he discovered later being purely arbitrary. Hermann (1915) believes he has succeeded in separating the "active substance" of the corpus luteum and of the placenta as a specific chemical substance, having identical physiological properties. Hermann possibly obtained his so-called active substance in a purer state. After engaging in special research work along this line during the last two years, Morley expresses the opinion that up to the present time no ideal method of preparation has been formulated, and until that is accomplished, standardization of the product will not be attempted. Considering the newness of the subject the article concludes with quite an extensive bibliography.

---

**Cardiac Sequellae of Asphyxiating Gas.** Ch. Fiessinger, Le Prog. Med., Oct. 20, 1917, calls attention to the action of the newer gases employed by the Germans, probably containing cyanogen compounds. Bradycardia and syncope are the principal symptoms. In sudden intoxication he recommends bleeding and ethero-camphorated oil. Later, the gastric hyperaesthesia is treated with bismuth preparations and theobromide and small doses of caffeine (0.10-0.20) are used to support the heart.

